# A Theoretical Raman Spectra Analysis of the Effect of the Li_2_S and Li_3_PS_4_ Content on the Interface Formation Between (110)Li_2_S and (100)β-Li_3_PS_4_

**DOI:** 10.3390/ma18153515

**Published:** 2025-07-26

**Authors:** Naiara Leticia Marana, Eleonora Ascrizzi, Fabrizio Silveri, Mauro Francesco Sgroi, Lorenzo Maschio, Anna Maria Ferrari

**Affiliations:** Chemistry Department, University of Turin, 10125 Turin, Italy; eleonora.ascrizzi@unito.it (E.A.); fabrizio.silveri@unito.it (F.S.); maurofrancesco.sgroi@unito.it (M.F.S.); lorenzo.maschio@unito.it (L.M.)

**Keywords:** Raman spectroscopy, Li_2_S, β-Li_3_PS_4_, solid-state interface, DFT, all-solid-state batteries (ASSBs)

## Abstract

In this study, we perform density functional theory (DFT) simulations to investigate the Raman spectra of the bulk and surface phases of β-Li_3_PS_4_ (LPS) and Li_2_S, as well as their interfaces at varying compositional ratios. This analysis is relevant given the widespread application of these materials in Li–S solid-state batteries, where Li_2_S functions not only as a cathode material but also as a protective layer for the lithium anode. Understanding the interfacial structure and how compositional variations influence its chemical and mechanical stability is therefore crucial. Our results demonstrate that the LPS/Li_2_S interface remains stable regardless of the compositional ratio. However, when the content of both materials is low, the Raman-active vibrational mode associated with the [PS_4_]^3−^ tetrahedral cluster dominates the interface spectrum, effectively obscuring the characteristic peaks of Li_2_S and other interfacial features. Only when sufficient amounts of both LPS and Li_2_S are present does the coupling between their vibrational modes become sufficiently pronounced to alter the Raman profile and reveal distinct interfacial fingerprints.

## 1. Introduction

In the past decades, energy shortage problems have become increasingly serious, and the search for clean energy and batteries with high storage capacity, in addition to being economically viable, has become a main research focus [1]. High-energy-density batteries are considered to be one of the most efficient ways to store energy. Lithium–sulfur (Li–S) batteries are one of the most promising candidates for the next generation of batteries due to their characteristics of cost efficiency, natural abundance, environmental friendliness, and a high theoretical energy density of 2500 W h kg^−1^ (or 2800 W h L^−1^) [2]. Furthermore, all-solid-state lithium batteries (ASSLBs) have shown superior performance compared to liquid electrolyte batteries, as ASSLBs are more stable and less flammable. However, the interface problem between materials arises, where the formation of Li dendrites or by-products, in addition to interfacial defects, can hinder proper functioning [3].

In this scenario, the choice of solid electrolyte is important given the low fluidity and insufficient wettability of the ASSLBs. No matter how high the ionic conductivity of the solid electrolyte is, the distribution of active materials, solid electrolytes, and electrically conductive materials must be homogeneous in the electrodes to obtain solid-state cells with high levels of performance [4]. Among sulfide-type electrolytes, β-Li_3_PS_4_ (LPS) stands out for its high ionic conductivity (3.0 × 10^−2^ Scm^−1^ at 573 K) [5]. Unfortunately, the interface between LPS and the Li anode is not electrochemically stable, and the decomposition of the electrolyte into Li_2_S and Li_3_P affects Li transport, increasing cell resistivity. A possible solution for this problem is to insert a functional buffer layer between the LPS electrolyte and the Li anode, preserving the cell components and their properties. Li_2_S seems to be one of the best choices given its electrochemical inertness with respect to Li metal and the stability of the LPS/Li_2_S and Li_2_S/Li interfaces [6,7,8]. 

The Raman spectrum is a useful tool for identifying the chemical composition of materials and determining the degree of purity based on the appearance (disappearance) of the characteristic vibrational mode peaks of the product (reactant) after synthesis is complete. This non-invasive technique allows one to investigate the chemical nature of components in both ex situ and in situ conditions. In ASSLBs, Raman spectroscopy has also been employed to monitor the evolution of interfaces within practical electrode composites after or during electrochemical treatment [9,10,11]. In particular, the LPS/Li_2_S interface can be characterized through Raman spectroscopy, taking into account the effects of electrochemical cycling. Identifying characteristic peaks in Raman spectra is key to understanding whether precursors have reacted to form the LPS/Li_2_S interface and for determining whether undesired degradation products or Li_2_S_2_ clusters have been formed [12,13,14,15,16].

Therefore, in this paper, we theoretically discuss the Raman spectra for the (100) LPS and (110) Li_2_S surfaces and the interfaces formed by modulating the Li_2_S and LPS content in the heterostructure. For the first time, the simulated Raman spectra of β-LPS and Li_2_S surfaces have been reported. DFT hybrid calculations carried out with the CRYSTAL23 program [17] were applied in order to precisely characterize this stable interface, whose structure has been already described in a previous publication [6]. Specific spectral features will be identified and related to the atomic structure and the local environment (thickness of the slabs).

## 2. Materials and Methods

The density functional theory (DFT) approach was combined with the hybrid PBE0 functional [18,19] and all-electron basis set 6–11G [20], 86–311G* [21], and 85–211dG [22] for Li, S, and P, respectively, as implemented in the CRYSTAL program.The adopted computational approach, which was already applied in our previous studies [6,23], has provided structures and energies in good accordance with data available in the literature. The accuracy of the truncation criteria for the bi-electronic integrals, Coulomb, and HF exchange series was controlled by a set of five thresholds with values of [8, 8, 8, 8, 16]. The reciprocal space was sampled according to a sublattice with shrinking factor 8, corresponding to 25 independent k-points in the irreducible part of the Brillouin zone. The LPS/Li_2_S heterostructures are completely relaxed in order to avoid the appearance of imaginary frequencies caused by geometrical constraints.

Adhesion energy ($E_{adh}$) is a key parameter for evaluating the interaction and stability of interfaces between solid materials, calculated according to the following equation:

(1) $$E_{adh}=\;\frac{E_{interface}-{(E}_{Li2S}+E_{LPS})}{A}$$

where $E_{interface}\;$ is the total energy of the heterostructure, *A* is the surface area of the interface, and $E_{{Li}_{2}S}$ and $E_{LPS}$ are the total energies of the two structures relaxed at the lattice parameters defining the interface. All $E_{adh}$ values have been corrected for basis set superposition error (BSSE) using the counterpoise method [24]. The BSSE correction does not exceed +3.55 meV/Å^2^ for PBE0 and +2.95 meV/Å^2^ for MN15. Both components are therefore either stretched or compressed during the interface formation process, and the energy cost for this deformation defines the strain energy (per surface unit), $E_{strain}$, which has to be taken into account for a proper estimate of the overall stability of the composite. $E_{strain}$ was computed as

(2) $$E_{strain}({Li}_{2}S;LPS)=\frac{E_{\left({Li}_{2}S;LPS\right)}-E_{\left({Li}_{2}S;LPS\right)}^{'}}{2A}$$

where $E_{\left({Li}_{2}S;LPS\right)}^{\prime }$ is the energy of the fully relaxed LPS or Li_2_S.

As in previous studies [25,26], to better estimate noncovalent interactions involved in the interface formation, energy estimates have also been made by using the Minnesota hybrid functional (MN15) [27] by computing single-point energy calculations on the PBE0 optimized structures.

The vibrational frequencies at the Γ point were computed within the harmonic approximation by diagonalizing the mass-weighted Hessian matrix of the second derivatives of the total energy per cell with respect to the pair of atomic displacements in the reference cell. Once the vibrational frequencies are calculated, the Raman intensities and the simulated Raman spectra can also be obtained. A temperature of 298 K is considered, along with laser wavelengths selected to recreate experimental conditions, with 632.8 nm for LPS, 488.0 nm for Li_2_S, and 532.0 nm for the interfaces [28,29,30]. More details on the computational vibrational frequencies calculated with the CRYSTAL program can be found in Ref. [31].

## 3. Results and Discussion

### 3.1. Bulk and Isolated Surfaces

Before discussing the modifications in the Raman spectra of the β-LPS/Li_2_S heterostructures, we first examine the Raman spectra of the pristine bulk and surface structures, previously presented and characterized in Ref. [6]. To our knowledge, this is the first time the Raman spectrum of the (100) surface of β-LPS has been computed.

**Figure 1:**
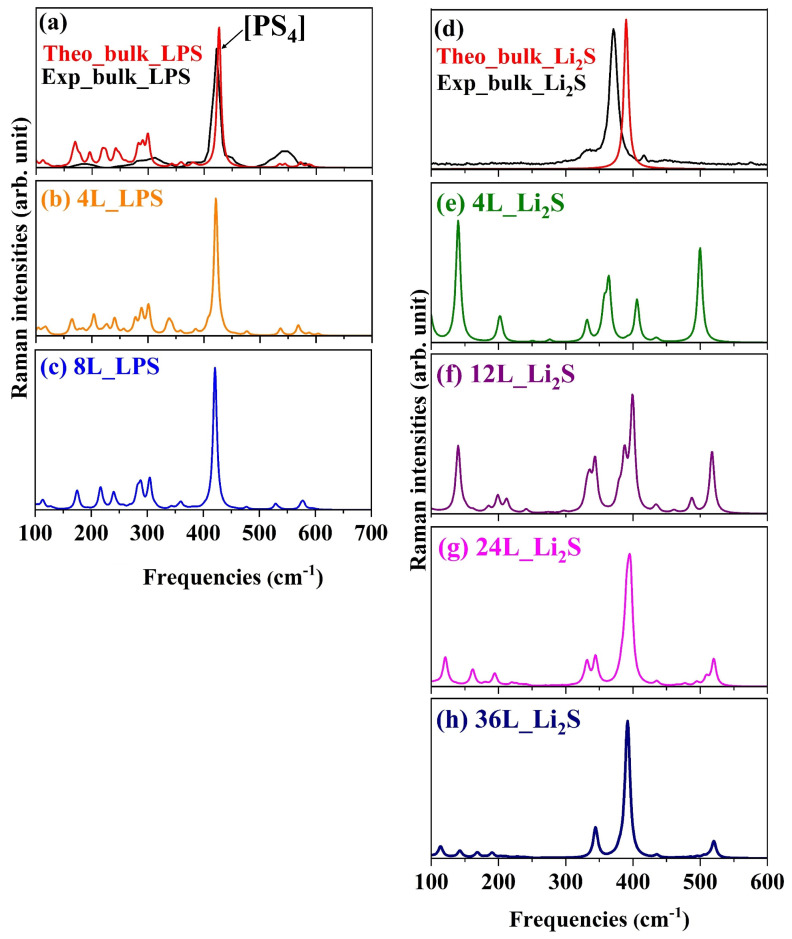
Computed Raman spectra of pristine materials at 298 K. Spectra were obtained using a wavelength of 632.8 nm for LPS and 488.0 nm for Li_2_S. Panels show (**a**) LPS bulk; (**b**) Li_2_S bulk; (**c**) and (**d**) LPS (100) surface with four and eight layers, respectively; (**e**–**h**) Li_2_S (110) surface with 4, 12, 24, and 36 layers, respectively. Experimental bulk spectra are reproduced from Refs. [28,29].

Figure 1a–c shows the Raman spectra of the bulk and (100) surface β-LPS with four and eight layers (4L and 8L). Both the general shape of the spectra and the position of the characteristic peak of the [PS_4_]^3−^ vibration at ~425 cm^−1^ agree well with previous experimental and theoretical studies on bulk LPS [12,15,28,30,32]. This characteristic peak presents similar intensities in both the bulk and surface structures. In the [PS_4_]^3−^ tetrahedron, the P–S bonds are covalent, and the overall structure is only moderately affected by the surrounding chemical environment. This limited influence is also reflected in the corresponding Raman features, which exhibit minimal variation. In addition to the intense main peak at 425 cm^−1^, the Raman spectra of both bulk and surface LPS also exhibit several signals in the 500–600 cm^−1^ range. These features are associated with vibrational modes involving the P–S within the PS_4_ tetrahedral units. At lower frequencies, additional peaks are observed, which are primarily attributed to the vibrational contributions of Li–S bonds present in the broader framework of the material. The complete description of vibrational modes, symmetry, and intensity are shown in Table S1.

When comparing the calculated Raman spectrum of bulk LPS with the experimental spectrum presented in Figure 1a, it is essential to emphasize that the peaks observed in the theoretical spectrum result from an idealized, perfectly ordered, and defect-free simulated structure. Conversely, the experimental sample exhibits a certain degree of structural disorder and defects (or are related to impurities during the synthesis), which leads to the broadening and attenuation of these peaks, making them less distinct and more challenging to resolve.

The Raman spectrum for the bulk Li_2_S has also been widely discussed in several articles in the literature [12,33], but to our knowledge, the experimentally acquired Raman spectrum for the (110) Li_2_S surface has not been reported. In contrast to LPS, Li_2_S exhibits a more pronounced difference between bulk and surface Raman spectra. The Raman spectrum of the bulk structure is characterized by a single peak at approximately 389 cm^−1^, which corresponds to Li–S stretching vibrations, as shown in Figure 1c. This well-defined feature reflects the high symmetry and long-range order of the crystalline bulk material. At the surface, however, the situation changes significantly. The reduced symmetry and increased degrees of freedom for the Li–S bonds lead to a splitting of at 389 cm^−1^ and the appearance of additional low-intensity peaks, particularly at lower and higher frequencies. These spectral changes are a direct consequence of structural rearrangements and local distortions confined to the outermost layers. As the number of layers increases, the Raman spectrum of the surface begins to resemble that of the bulk: the peaks gradually merge and become more defined, indicating a progressive restoration of the bulk-like crystallinity (compare the spectra reported in Figure 1e–h). For example, in the 36L_Li_2_S system, Figure 1h, the original 389 cm^−1^; peak is split into three components (at 390, 391, and 392 cm^−1^), and additional features appear around 344 cm^−1^ and 520 cm^−1^. These are attributed to S–Li–S bending, Li–S stretching, and mixed modes activated by surface symmetry breaking and to the Li–S–Li vibrational modes involving mainly the topmost surface layers (see Table S2 for more details).

### 3.2. LPS/Li_2_S Heterostructure

After having identified the characteristic Raman peaks of each material and their isolated surfaces, we can proceed to analyze the LPS/Li_2_S heterostructures. As previously discussed, these materials are widely employed in sulfur-based solid-state lithium batteries. Their interface commonly arises either at the cathode/solid-electrolyte boundary [34] or when Li_2_S is used as a protective layer for the lithium anode in contact with LPS [6,7,8]. In this context, understanding and identifying the features of the interface is essential to evaluating the behavior and stability of the system. Previous studies [6] conducted by our group have examined the LPS/Li_2_S interface particularly under conditions where only a limited amount of Li_2_S was introduced (4L of Li_2_S was considered). The results showed that the interface is chemically and mechanically stable, indicating a good intrinsic compatibility between the two materials.

Based on this, we decided to analyze the same interface (100)LPS/(110)Li_2_S, its stability, and its Raman spectrum profile, taking into account different amounts of LPS and Li_2_S. We simulated the following interfaces:

(1) 8L of LPS with 12L of Li_2_S (8L_LPS/12L_Li_2_S);
(2) 4L of LPS with 12L of Li_2_S (4L_LPS/12L_Li_2_S);
(3) 4L of LPS with 24L of Li_2_S (4L_LPS/24L_Li_2_S);
(4) 4L of LPS with 36L of Li_2_S (4L_LPS/36L_Li_2_S);
(5) 8L of LPS with 36L of Li_2_S (8L_LPS/36L_Li_2_S).

**Figure 2:**
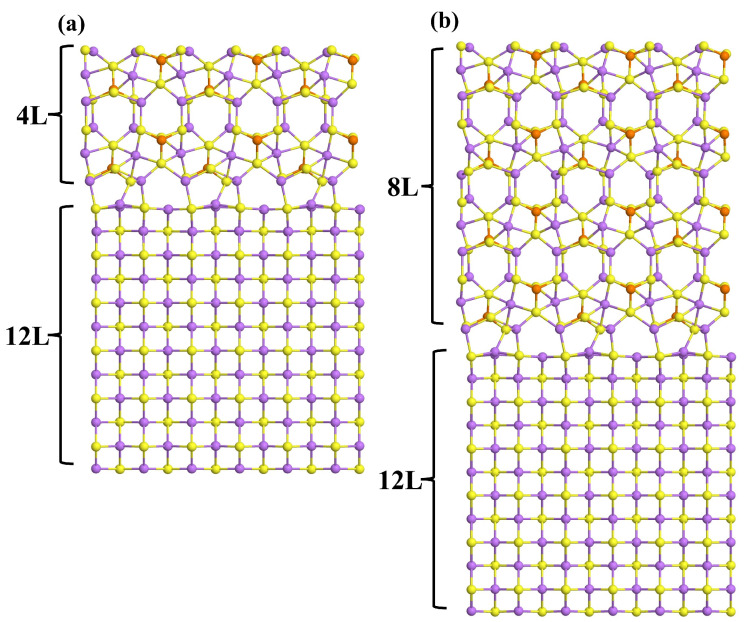
(100)LPS/(110)Li_2_S interfaces: (**a**) 4L_LPS/12L_Li_2_S and (**b**) 8L_LPS/12L_Li_2_S. Lithium, sulfur, and phosphorus atoms are represented as purple, yellow, and orange spheres, respectively.

The optimized interface models are shown in Figure 2 and Figure S1, and their adhesion energies are shown in Table 1. According to our results, the content of Li_2_S and LPS at the interface has little effect on its chemical and mechanical stability, the *E_adh_* values are between –27 and –31 meV/Å^2^, and only slight structural changes are observed, mostly confined to the interfacial region, with the non-interfacial layers remaining largely unaffected. The 4L_LPS/36L_Li_2_S and 8L_LPS/36L_Li_2_S models present a reduction in ~1 meV/Å^2^ in *E_adh_*, which may be due to the high structural stability of Li_2_S that is mostly undisturbed by the presence of LPS and that recovers its bulk structure after the first 12 layers. Although the MN15 functional yields significantly larger energy values (ranging from –47 to –50 meV·Å^−2^), the predicted stability order of the heterostructures remains consistent with that computed using the PBE0 functional. The stability of the heterostructures can be assed evaluating the work of adhesion [35,36,37], *W_adh_ =* –(*E_adh_ + E_strain_*). *W_adh_* quantifies the energy required to separate the heterostructure into two isolated slabs, accounting for both the adhesion energy and the strain induced by heterostructure formation. In all the heterostructure examined in this work, *E_strain_* is only a fraction of the *E_adh_*, indicating thermodynamic stability. As a result, a positive *W_adh_* must be supplied to separate the interfaces. Although the computed *E_adh_* may appear relatively small, it is important to consider that such heterostructures are likely to form under experimental conditions, as the synthesis and stability of the interface in the device involves the application of external pressure [38,39].

**Figure 3:**
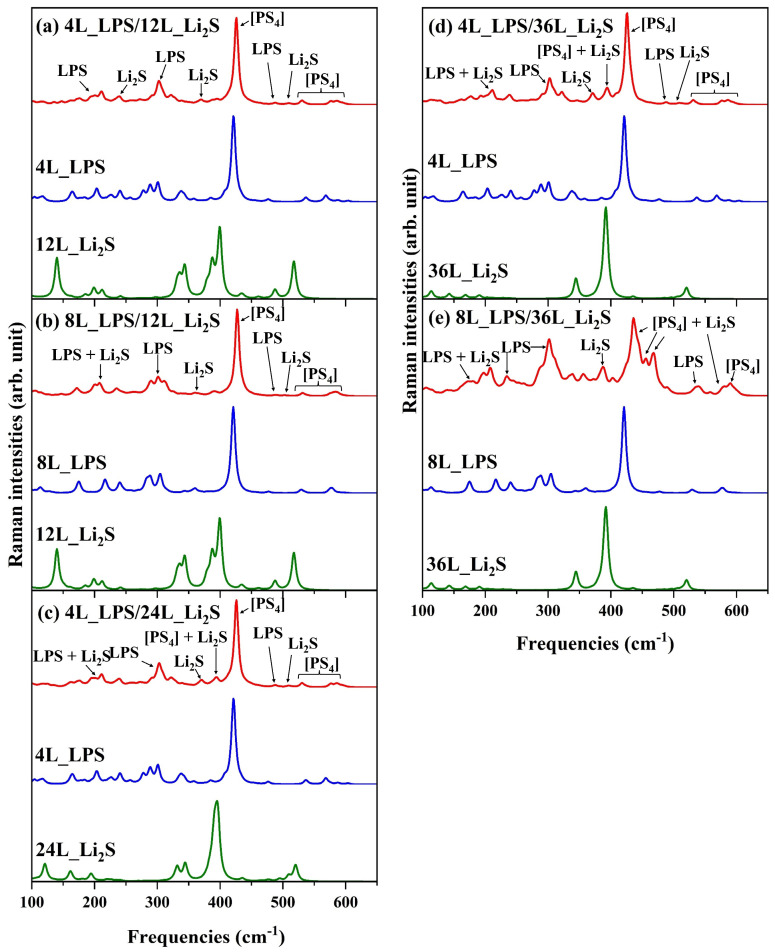
Raman spectra of the interface and the respective pristine surfaces at 298 K and a wavelength of 632.8 nm for LPS, 488.0 nm for Li_2_S, and 532.0 nm for the interfaces: (**a**) 4L_LPS/12L_Li_2_S, (**b**) 8L_LPS/12L_Li_2_S, (**c**) 4L_LPS/24L_Li_2_S, (**d**) 4L_LPS/36L_Li_2_S, and (**e**) 8L_LPS/36L_Li_2_S.

From now on, we will analyze the simulated Raman spectra of all interfaces. Figure 3 contains the Raman spectra for the heterostructures and, for the sake of comparison, the pristine surfaces. As can be seen in Figure 3, the characteristic peak related to [PS_4_]^3−^ of LPS appears in all the Raman spectra regardless of LPS content. The intensity of this peak is not significantly affected by the formation of the interface, although a slight shift in its position is observed. In general, the vibrational peaks associated with LPS remain identifiable in the Raman spectra of the interface, showing only subtle changes in both intensity and frequency compared to the pristine LPS surface. These variations can be attributed to the relative LPS content within the interface region.

It is important to note that the vibrational modes related to LPS—particularly those associated with the [PS_4_]^3−^ tetrahedral unit—exhibit much higher intensities than those originating from the Li_2_S surface. As the Li_2_S content increases, however, more prominent and partially degenerate Li_2_S-related peaks begin to emerge. This effect is clearly illustrated by comparing the spectra of the 8L_LPS/4L_Li_2_S and 8L_LPS/12L_Li_2_S heterostructures (see Figure S2 and Figure 3b). In the latter, characteristic Li_2_S peaks become distinguishable at approximately 365 cm^−1^, 392 cm^−1^, and 511 cm^−1^, corresponding to Li–S bond stretching modes. This observation aligns with previous reports highlighting the experimental challenges in detecting Li_2_S during the synthesis of Li_3_PS_4_. In such cases, Raman measurements often fail to capture Li_2_S-related peaks following the formation of Li_3_PS_4_, likely due to their lower intensity and possible overlap with LPS signals [40].

In our case, significant modifications to the Raman spectra are observed for the 8L_LPS/36L_Li_2_S interface, where both LPS and Li_2_S are present in high concentrations. Under these conditions, the main LPS peak couples with the stretching vibrational modes of Li_2_S, resulting in the formation of three distinct peaks characteristic of the interface at 435 cm^−1^, 456 cm^−1^, and 468 cm^−1^. Additionally, typical Li_2_S vibrations appear at the interface as a split peak at 388 cm^−1^, a broad band around 300 cm^−1^ due to the overlap of Li–S stretching modes from both Li_2_S and LPS, and a distinct peak at 558 cm^−1^. Notably, the region between 570 cm^−1^ and 590 cm^−1^ continues to display LPS-related vibrations, with no significant contribution from Li_2_S modes. The details of the peaks and their descriptions are reported in Table S3 and discussed in Section S1 of Supplementary Information. The broadening and splitting of the peak around ~450 cm^−1^ can thus be regarded as a distinctive spectroscopic fingerprint indicative of the LPS/Li_2_S interface reflecting changes in local bonding environments and structural heterogeneity. The simulated spectrum likely represents the most accurate model of what would be observed experimentally in a well-formed interface with a balanced composition, providing an important reference for interpreting Raman data. This highlights the importance of detailed spectral analysis, which is essential not only for elucidating how the relative amounts of Li_2_S and LPS influence the Raman spectrum but also for enabling a reliable interpretation of the experimental results.

Li_2_S may be intentionally introduced to promote the formation of a stable interface with LPS, where it plays a beneficial role as a passivating coating that prevents the decomposition of LPS in contact with a lithium metal anode. However, Li_2_S can also arise as an unintended residual byproduct from the synthesis of LPS. In this context, residual Li_2_S impurities have been shown to adversely affect the ionic conductivity of the solid electrolyte, thereby compromising lithium metal battery performance by increasing overpotentials and shortening cycle life.

Therefore, understanding and controlling the interfacial composition through spectroscopic evaluation is essential for optimizing solid-state battery performance. According to Mirmira et al. [30], Raman spectroscopy could detect residual Li_2_S in LPS samples when the impurity concentration was above 12 mol%. However, for concentrations below this threshold, Raman spectroscopy’s sensitivity diminished, making it challenging to identify smaller amounts of Li_2_S impurities. They found that until 30 mol% of Li_2_S in the sample, the peaks related to the Li-S vibration of Li_2_S (at ~380 cm^−1^) are evident, and then the intensity decreases with the Li_2_S concentration due to the higher intensity of LPS vibrations. The same peak was also evident in the Li_2_S-LPS composite obtained by Jiang and co-workers [39]. The composite structure was evaluated by Raman spectroscopy and XRD diffraction, and the authors observed that when the lithium sulfide in the starting material was excessive, such as a 45% and 62.5% mass fraction of Li_2_S, the phase LPS was generated with a residual lithium sulfide, and the characteristic peaks related to LPS (at 420 cm^−1^ related to [PS_4_]^3−^ cluster) and Li_2_S (at 380 cm^−1^ attributed to Li–S–Li vibration) were apparent in the Raman spectra of the composite. Thus, the presence of these signals does not necessarily indicate the formation of a stable interface but may instead correspond to residual impurities and, even more critically, residual Li_2_S may be present below the detection limit of Raman spectroscopy, making its presence difficult to identify and potentially leading to underestimated impurity levels that can adversely affect battery performance.

## 4. Conclusions

In this study, we performed a theoretical Raman spectra analysis to investigate the effect of Li_2_S and Li_3_PS_4_ content on the interface formation between (110) Li_2_S and (100) β-Li_3_PS_4_. Our results confirm that the LPS/Li_2_S interface remains stable regardless of its content. However, we observed that a higher concentration of LPS complicates the spectral identification of the interface, as the intense vibrational peak of the [PS_4_]^3−^ cluster overshadows the characteristic Li_2_S peaks and other interface-related signals.

The analysis of heterostructures with different LPS and Li_2_S contents showed that, while small amounts of Li_2_S induce minimal modifications in the Raman spectrum, a significant increase in Li_2_S leads to substantial spectral changes. Notably, the broadening and splitting of the peak at ~450 cm^−1^, attributed to the coupling of [PS_4_]^3−^ and Li_2_S vibrations, can be considered a spectral fingerprint of the formed interface. This feature was only observed at higher amounts of LPS and Li_2_S, suggesting its importance for identifying the interface experimentally.

Our findings provide valuable insights into the Raman spectral characteristics of LPS/Li_2_S interfaces, emphasizing the need for careful spectral interpretation, especially when working with low Li_2_S content. This study contributes to the understanding of interface formation in all-solid-state lithium batteries, which is key for optimizing their performance and stability.

## Figures and Tables

**Table 1:** *E_adh_* and *E_strain_* (meV/Å^2^) of all interface models computed as in Equations (1) and (2). a and b represent the cell parameters of the surfaces and interfaces.

	*a*	*b*	*E_strain_* (Li_2_S)	*E_strain_* (LPS)	$E_{adh}$	$E_{adh}$
PBE0						MN15
**(110)Li_2_S**	5.70	8.06	-	-	-	
**(100)LPS**	6.23	8.28	-	-	-	
**4L_LPS/12L_Li_2_S**	5.82	8.04	1.39	7.36	−29.15	−48.32
**8L_LPS/12L_Li_2_S**	5.88	8.05	4.03	5.29	−28.79	−47.81
**4L_LPS/24L_Li_2_S**	5.82	8.04	0.33	7.45	−27.76	−50.54
**4L_LPS/36L_Li_2_S**	5.82	8.04	1.02	7.50	−31.23	−48.99
**8L_LPS/36L_Li_2_S**	5.88	8.06	0.74	5.34	−29.12	−47.82

## Data Availability

The original contributions presented in this study are included in the article/Supplementary Materials. Further inquiries can be directed to the corresponding authors.

## Supplementary Materials

The following supporting information can be downloaded at: https://www.mdpi.com/article/10.3390/ma18153515/s1, Figure S1: (100)LPS/(110)Li_2_S interfaces with different LPS and Li2S contents: (a) 4L_LPS/24L_Li_2_S, (b) 4L_LPS/36L_Li_2_S, and (c) 8L_LPS/36L_Li_2_S; Figure S2: Raman spectra of the interface 8L_LPS/4L_Li_2_S and the respective pristine surfaces; Table S1: Vibrational mode descriptions of β-Li_3_PS_4_ bulk and its (100) surface; Table S2: Vibrational mode descriptions of Li_2_S bulk and its (100) surface; Table S3: Vibrational mode descriptions of the interface 8L_LPS/36L_Li_2_S; Discussion about the Raman spectra of the 8L_LPS/36L_Li2S interface compared with their isolated components.
