# Supplementary material for: A Theoretical Raman Spectra Analysis of the Effect of the Li2S and Li3PS4 Content on the Interface Formation Between (110)Li2S and (100)β-Li3PS4
Source: Materials (Basel). 2025 Jul 26;18(15):3515. doi: 10.3390/ma18153515 (PMC12347676; doi:10.3390/ma18153515)
Supplement: Supplementary file 1 [file materials-18-03515-s001.zip › materials-3736575-supplementary.pdf]

Supplementary Materials

A Theoretical Raman Spectra Analysis of the Effect of the Li_2S and Li_3PS_4 Content on the Interface Formation between (110)Li_2S and (100)β-Li_3PS_4

Naiara Leticia Marana *, Eleonora Ascrizzi, Fabrizio Silveri, Mauro Francesco Sgroi, Lorenzo Maschio and Anna Maria Ferrari *

Chemistry Department, University of Turin, 10125, Turin, Italy; eleonora.ascrizzi@unito.it (E.A.); fabrizio.silveri@unito.it (F.S.); maurofrancesco.sgroi@unito.it (M.F.S.); lorenzo.maschio@unito.it (L.M)

* Correspondence: naiaraleticia.marana@unito.it (N.L.M.); anna.ferrari@unito.it (A.M.F.)

Supplementary Information

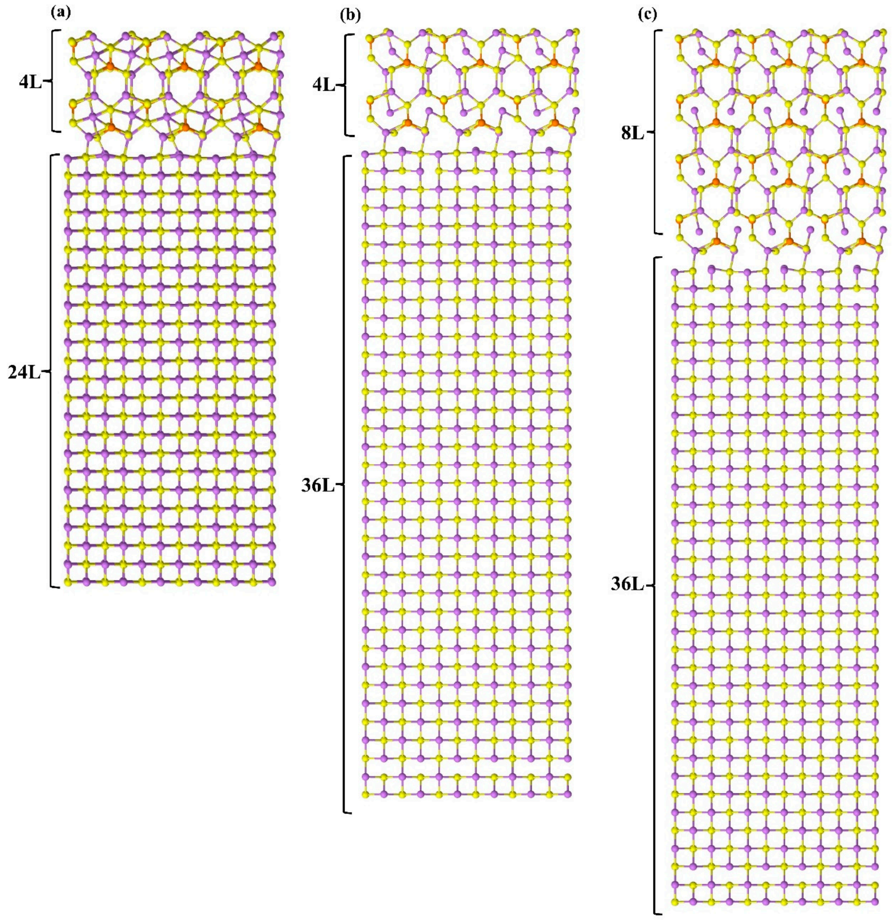

Figure S1. (100)LPS/(110)Li_2S interfaces with different LPS and Li2S contents: (**a**) 4L_LPS/24L_Li_2S, (**b**) 4L_LPS/36L_Li_2S, and (**c**) 8L_LPS/36L_Li_2S.

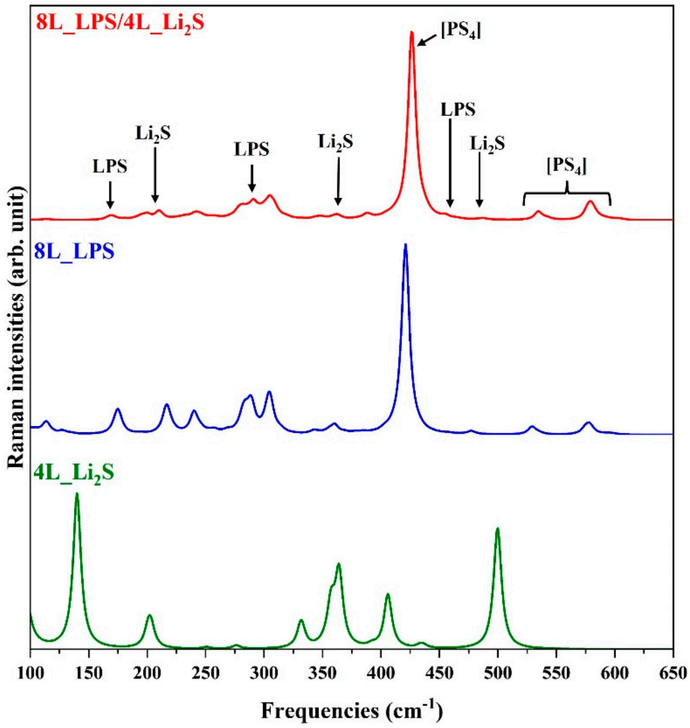

Figure S2. Raman spectra of the interface 8L_LPS/4L_Li$_2$S and the respective pristine surfaces.

Table S1. Vibrational mode descriptions of β-Li_3PS_4 bulk and its (100) surface.

Frequency (cm^{-1})	Symmetry	Intensity	Frequency (cm^{-1})	Symmetry	Intensity	Description
LPS_bulk			LPS_8L			
165.5801	A1	13.23				
170.0978	A2	31.6				
178.289	A1	14.09	174.9028	A1	31.26	
196.165	B2	26.22				bending and stretching vibration motion Li-S and S-Li-S (Li-S-Li)
224.6416	B1	28.53	218.1643	B1	55.13	
241.6964	A1	43.38	240.8395	A1	69.26	
247.4099	A2	16.16				
282.2609	A1	64.64	284.6175	B1	49.66	
290.2571	B2	52.34	290.4926	A1	54.21	Li-S vibration coupled to movement of [PS4] cluster
299.6924	A1	126.79	303.1897	A1	83.03	
360.0752	B2	16.21	361.9486	A1	30.11	movement becomes symmetrical on surface with greater intensity in atoms of outer layers
426.6282	A1	1000	422.1148	A1	1000	slight displacement of characteristic peak of [PS4] cluster on surface
534.7558	A1	31.3	530.0517	A1	54.18	
544.5723	A2	32.93	575.5609	A1	32.32	symmetrical vibrational motion of P-S divided into three components (x, y, z)
572.1023	A1	45.79	580.1072	A1	45.7	
587.1970	B2	19.19	581.2779	B1	27.36	asymmetrical vibrational motion of P-S

Table S2. Vibrational mode descriptions of Li_2S bulk and its (100) surface.

Frequency (cm⁻¹)	Symmetry	Intensity	Frequency (cm⁻¹)	Symmetry	Intensity	Description
**Li_2S_bulk**			**Li_2S_36L**			
			14.2448	Ag	24.81	S–Li–S bending, Li–S stretching, and mixed modes activated by surface symmetry breaking
			50.4717	Ag	23.11	
			82.8949	Ag	22.92	
			113.8876	Ag	22.59	
			142.5374	Ag	21.75	
			168.554	Ag	21.98	
			190.6785	Ag	23.55	
			344.3468	Ag	404.26	
389.8488	F2g	1000.00	390.3006	B3g	523.30	at the surface, peak is slightly shifted and vibrational motion is divided not only into x, y, and z components but also into larger motion of inner (~390cm^{-1}) and outer (~392cm^{-1}) atoms
			391.6407	Ag	642.40	
			392.8148	B3g	1000	
			435.5194	Ag	50.56	activated internal bending and stretching modes of S^{2-} anions due to surface symmetry breaking
			484.6309	Ag	22.37	
			495.4429	Ag	32.9	
			505.4229	Ag	43.64	
			520.3345	Ag	397.45	Li-S-Li bending for outermost layers

Table S3. Vibrational mode descriptions of the interface 8L_LPS/36L_Li_2S.

Frequency (cm⁻¹)	Symmetry	Intensity	Frequency (cm⁻¹)	Symmetry	Intensity	Frequency (cm⁻¹)	Symmetry	Intensity
**8L_LPS/36L_Li_2S**			**36L_Li_2S**			**8L_LPS**		
57.054	A	3.89	50.4717	Ag	23.11			
92.3603	A	6.09	82.8949	Ag	22.92			
106.0552	A	8.17	113.8876	Ag	22.59			
144.6978	A	9.9	142.5374	Ag	21.75			
197.5733	A	26.15				174.9028	A1	31.26
207.5631	A	125.37				218.1643	B1	55.13
233.4264	A	17.57	190.6785	Ag	23.55			
233.6509	A	40.34						
246.8415	A	15.15				240.8395	A1	69.26
261.1898	A	13.63						
284.5554	A	22.98				284.6175	B1	49.66
301.9027	A	223.65				290.4926	A1	54.21
311.0452	A	130.04				303.1897	A1	83.03
333.6052	A	46.51						
339.3448	A	115.42	344.3468	Ag	404.26			
354.6664	A	60.01				361.9486	A1	30.11
371.9686	A	45.98						
387.7918	A	119.76	392.8148	B3g	1000			
402.5737	A	147.35						
406.7944	A	61.62						
424.4032	A	215.63				422.1148	A1	1000
435.6214	A	1000	435.5194	Ag	50.56			
443.8009	A	264.14						
456.3922	A	257.11						

468.8379	A	267						
489.9682	A	90.12	484.6309	Ag	22.37			
541.455	A	71.76	495.4429	Ag	32.9	530.0517	A1	54.18
558.1156	A	60.6	505.4229	Ag	43.64	575.5609	A1	32.32
581.9106	A	63.02	520.3345	Ag	397.45	580.1072	A1	45.7
590.5283	A	166.91				581.2779	B1	27.36

Discussion about the Raman spectra of the 8L_LPS/36L_Li_2S interface compared with their isolated components

The vibrational spectrum reveals distinct features that differentiate the interface between LPS and Li_2S from individual systems. In the low-frequency region (100–150 cm^{-1}), the interface displays a significantly higher density of vibrational modes compared to either LPS or Li_2S alone. These low-energy modes are indicative of collective lattice motions, particularly when involving lithium ions. Their enhancement at the interface suggests increased ionic flexibility and lattice softening, likely due to structural distortions and symmetry breaking introduced at the interfacial region. Between 150 cm^{-1} and 300 cm^{-1}, the LPS spectrum shows features associated with vibrations of the $[PS_4]^{3-}$ cluster, while Li_2S contributes fewer but more localized modes. The 380–500 cm^{-1} region of the interface spectrum reveals broadened, shifted, and new vibrational modes arising from the distortion of the $[PS_4]^{3-}$ cluster, the coupling of P-S and Li-S stretching vibrations, and the emergence of unique local bonding environments, indicating real chemical interactions and a combination between the vibrational modes of LPS and Li_2S, which is also due to the proximity of S^{2-} and $[PS_4]^{3-}$ anions. In the 400–500 cm^{-1} region, characteristic internal modes of the $[PS_4]^{3-}$ units are prominent in LPS and are partially retained in the interface. A distinct Li_2S signature near ~390 cm^{-1} (of isolated Li_2S) also appears in the interface spectrum (386–406 cm^{-1}), suggesting the preservation of structural modes from both materials. Above 500 cm^{-1}, weaker modes emerge in the interface spectrum, which may correspond to combination or higher-order vibrational features resulting from anharmonic coupling and local structural distortions, further confirming the formation of unique bonding environments not present in the isolated materials (~545 cm^{-1}). Finally, in the 560–600 cm^{-1} range, the interface spectrum shows a splitting of the vibrational mode that appears as a single peak in LPS, suggesting that the local symmetry of the $[PS_4]^{3-}$ cluster is broken at the interface, giving rise to two distinct vibrational components.
